# Blueberry Supplementation and Skin Health

**DOI:** 10.3390/antiox12061261

**Published:** 2023-06-12

**Authors:** John Ivarsson, Alessandra Pecorelli, Mary Ann Lila, Giuseppe Valacchi

**Affiliations:** 1 Plants for Human Health Institute, Department of Food, Bioprocessing, and Nutrition Sciences, North Carolina State University, Kannapolis, NC 28081, USA; jcivarss@ncsu.edu (J.I.J.); mlila@ncsu.edu (M.A.L.); 2 Department of Environmental Sciences and Prevention, University of Ferrara, 44121 Ferrara, Italy; apecore@ncsu.edu; 3 Regenerative Medicine, Department of Animal Science, North Carolina State University, Kannapolis, NC 28081, USA; 4 Department of Food and Nutrition, Kyung Hee University, Seoul 02447, Republic of Korea

**Keywords:** blueberries, skin, inflammation, polyphenol, antioxidant

## Abstract

Environmental stressors such as air pollutants, ozone, and UV radiation are among the most noxious outdoor stressors affecting human skin and leading to premature skin aging. To prevent the extrinsic aging, the skin is equipped with an effective defensive system. However, cutaneous defense mechanisms can be overwhelmed through chronic exposure to environmental pollutants. Recent studies have suggested that the topical usage of natural compounds, such as blueberries, could be a good strategy to prevent skin damage from the environment. Indeed, blueberries contain bioactive compounds found to induce an active skin response against the environmental noxious effects. In this review, results from recent studies on this topic are discussed in order to build the argument for blueberries to possibly be an effective agent for skin health. In addition, we hope to highlight the need for further research to elucidate the mechanisms behind the use of both topical application and dietary supplementation with blueberries to bolster cutaneous systems and defensive mechanisms.

## 1. Introduction

Blueberries contain a broad profile of phytochemicals associated with a variety of human health benefits. The main mechanism by which environmental stressors affect the skin is via the generation of reactive oxygen species (ROS) [1]. Excess ROS might overwhelm the cutaneous defense mechanisms causing oxidative stress that can then result in premature aging. The present work highlights the state-of-the-art on the ability of blueberry to protect cutaneous systems from environmental stressors through both topical application of blueberry-derived products and dietary consumption.

## 2. Cutaneous Anatomy and Physiology

Human skin (~2 m^2^) is composed of several distinct layers which house different physiological systems and subsequent functions. The epidermis, the most superficial layer of the skin, can be subdivided into five layers based upon keratinocytes differentiation: stratum corneum, stratum lucidum, stratum granulosum, stratum spinosum, and stratum basale [2]. The outermost layer, stratum corneum, is the principal barrier layer, consisting of a complex arrangement of terminally differentiated keratinocytes, defined as corneocytes, embedded in a peculiar lipid matrix mainly composed of cholesterol, fatty acid, and ceramides [2]. This complex structure, first described as a “brick and mortar system”, creates a tortuous pathway and barrier for cutaneous stressors [3,4]. These layers represent the first line of defense against physical trauma, microorganisms, and environmental stressors such as ultraviolet radiation, particulate matter, and ozone [5]. The second layer of the skin, the dermis, is directly connected to the epidermis and can be segmented into two layers. The papillary layer, stratum papillare, connects the dermis to the epidermis and consists of thin collagen fibers; the reticular layer, stratum reticulare, is the deepest layer of the dermis and is composed of dense collagen fibers [6,7]. The dermis is composed of numerous cellular components: principally fibroblasts, along with macrophages, T and B cells, mast cells, blood vessels, lymphatics, and nerves [5,7]. The hypodermis, also known as the subcutaneous layer, is the final layer of the skin, which is composed of adipose tissue. This thick lipid layer is particularly useful in its ability to act as a barrier, store water, and absorb various lipophilic compounds [2,6]. The skin is vital for protection, thermoregulation, sensation, water storage, absorption, expression, and synthesis of vitamin D_3_ [8].

Aside from the lipid matrix barrier, the skin is equipped with several enzymatic and non-enzymatic defensive mechanisms. Endogenous defensive enzymes prevent a harmful accumulation of ROS such as the superoxide anion (O_2_^•−^), hydroxyl radical (OH^•^), alkoxy radical (RO^•^), and peroxyl radical (ROO^•^) [9]. Catalase (CAT), superoxide dismutase (SOD), and glutathione peroxidase (GPX) are three enzymes present in the skin which are responsible for preventing cutaneous oxidative damage [10]. This physiological mechanism can handle moderate levels of ROS; however, exposure to environmental pollutants can result in an excessive and chronic increase in ROS which overwhelms the antioxidant defenses of the skin [5,11]. Levels of these endogenous antioxidant enzymes vary between the epidermis and the dermis, with a higher concentration in the epidermis than dermis [5].

In addition to this enzymatic system, numerous nonenzymatic antioxidant micronutrients also help to prevent oxidative damage. L-ascorbic acid (vitamin C), ɑ-tocopherol (vitamin E), glutathione (GSH), uric acid, and ubiquinol are among the most predominant cutaneous antioxidants [12]. Vitamin E is a lipid-soluble antioxidant that protects the skin by preventing lipid peroxidation [13]. Furthermore, increased levels of vitamin E have been shown to inhibit prostaglandin E2 (PGE2) expression, a key mediator of inflammation linked to skin aging [13,14]. In addition to vitamin E, vitamin C also plays a key protective role [5,15,16]. Of note, the epidermis houses more of these antioxidants than the dermis in a gradient fashion, with the highest levels in the epidermis deeper layers and the lower levels in the stratum corneum [12].

## 3. Principal Environmental Stressors to Cutaneous Systems

Ultraviolet light (UV) exposure is necessary for our body to produce vitamin D_3_. However, prolonged exposure can result in a cascade of harmful cutaneous effects [17]. UV light represents wavelengths of 400 nm to 100 nm and can be divided into UVA and UVB. UVA radiation consists of 400–314 nm wavelengths and represents more than 95% of UV light which passes through earth’s protective stratospheric ozone layer. UVB radiation is highly mutagenic and cytotoxic to cutaneous systems; however, it is usually absorbed efficiently by the ozone layer [18]. UV light absorption causes the photochemical generation of ROS, which leads to a cascade of damaging processes in cutaneous structures [19]. While ROS are naturally produced in skin cell mitochondria during normal oxidative metabolism, the continued production from exogenous stressors such as UV light, pollution (including ozone, particulate matters, etc.), and cigarette smoke can overwhelm antioxidant defenses, resulting in oxidative damage and contributing to skin extrinsic premature aging [20,21]. Indeed, as previously mentioned, although the skin is well equipped with enzymatic and non-enzymatic defensive systems, the continued chronic exposure to outdoor insults can overcome the cutaneous physiological protection and lead to skin damage [22]. 

Tropospheric ground level ozone (O_3_) is the result of photochemical reactions between nitrogen oxides, volatile organic compounds, and carbon monoxide [23]. The sources of these compounds are pollutants emitted from power plants, cars, and chemical plants. In the United States, over 100 million people reside in areas that exceed the health-based National Ambient Air Quality Standard (NAAQS) of 70 parts per billion (ppb) for O_3_ [24]. This statistic becomes more daunting when using the World Health Organization (WHO) O_3_ standards, which are set at 50 ppb [25]. Daily exposure to high concentrations of O_3_ has been associated with higher incidence of respiratory and cardiorespiratory mortality, especially in those with preexisting chronic conditions such as asthma [26]. Unlike UV light, O_3_ is unable to penetrate the cutaneous tissues, and its effect is mainly mediated by a cascade of bioactive reactions, leading to increased lipid peroxidation and ROS formation [27] which modulate key physiological inflammatory pathways and exhaust cutaneous antioxidants, causing adverse skin conditions [28,29]. Indeed, although ozone cannot penetrate skin, it can initiate free radical reactions by interacting with biomolecules present within the outermost layer of the skin, stratum corneum (SC), including lipids, proteins, and DNA, leading to the production hydrogen peroxide (H_2_O_2_) and nonradical species, such as aldehydes [30]. O_3_ secondary mediators can further perpetuate the damage throughout the skin by interacting with keratinocytes and fibroblasts, inducing oxidative stress reactions and lipid peroxidation [31].

Particulate matter (PM) is air pollution that is a mixture of solid and liquid particles of varying sizes. These particles can contain acids, organic chemicals, metals, soils, or dust particles and are categorized by diameter (µM): PM_10_, PM_2.5_, and PM_0.1_. Sources of PM include natural origins such as volcanoes, dust storms, and forest fires; industries contribute to PM levels through processing, burning of fossil fuels, and industrial waste products. Epidemiological studies have associated 8.9 million deaths in 2015 with long-term exposure to fine PM [32]. Fine–ultrafine PM_0.1–2.5_ is most directly responsible for skin barrier dysfunction and the exacerbation of skin ailments such as atopic dermatitis, due to their ability to circumvent physiological barriers [33]. The mechanisms involved in PM-associated skin disorders result from increased oxidative damage due to PM interaction with the skin cells. Although still under debate, it has been suggested that PMs can move through the skin through hair follicles or transdermally, generating a cascade of biological oxidative damage stress. PAHs are components of UFPs that can be absorbed through the skin and eventually damage the mitochondria, resulting in intracellular ROS production. These damaged mitochondria produce superoxide anions, which can be converted into H_2_O_2_ that can then undergo the Fenton reaction to produce hydroxyl radicals, resulting in increased ROS and activation of redox sensitive transcription factors, such as AP1 and NFκB. In addition, interactions between PM particles and surfaces can result in extracellular ROS production, again resulting in the activation of redox-sensitive transcription factors AP1 and NFκB. The consequences of oxidative stress result in antioxidant depletion, lipid peroxidation, and DNA damage. In support of this idea, our lab has demonstrated that exposure to PM particles induces nuclear translocation of NFκB, increases levels of HNE, and promotes DNA damage in ex vivo human biopsies [34,35].

## 4. Blueberry Phytoactives

Blueberries contain an abundance of bioactive compounds such as flavonoids and other polyphenolic compounds, which have strong antioxidant, anti-inflammatory, immunomodulatory, antimicrobial, and other health-relevant properties [36]. The polyphenolic composition of blueberries is influenced by several factors such as variety, season, method of cultivation, and growing location [37]. Anthocyanins are one of the predominant polyphenols present in blueberries which are responsible for their blue color; additionally, they are also one of the most powerful natural antioxidants [38]. In vitro studies exhibit anthocyanin’s significant antioxidative ability to scavenge ROS and have beneficial biological functions such as enzyme inhibition, as well as anti-inflammatory and antibacterial effects [39,40]. Consistent consumption of blueberries has been proven to increase total anthocyanin-derived metabolites in blood serum [41]. While anthocyanins are the dominant form of polyphenol in blueberries, they also contain many other phenolic compounds such as flavonols, ellagitannins, proanthocyanidins, hydroxycinnamic acids, gallotannins, and hydroxybenzoic acids [42]. Polyphenols undergo significant modification through the digestive process as they are metabolized; microbes biotransform polyphenols through deglycosylation, dihydroxylation, demethylation, decarboxylation, and isomerization, creating a variety of low molecular weight analytes which are either intermediates or products of metabolism. When the three phases of polyphenol digestion (salivary, gastric, and intestinal) are complete, 169 polyphenolic metabolites can be found in measurable amounts in blood plasma [42]. Among the 169 serum polyphenol metabolites, 58 blueberry-derived metabolites significantly change in concentration following blueberry consumption [42]. Table 1 exhibits the top five metabolites upregulated in blood plasma following the consumption of a blueberry beverage. While many question the ability of polyphenols to demonstrate antioxidant action in vivo because of their apparent poor bioavailability, the current literature suggests that the consumption of polyphenol-rich blueberries protects against gastrointestinal problems, cancers, diabetes, and cardiovascular and neurodegenerative diseases through their phenolic metabolites after catabolism by the gut microbiome [43,44]. Furthermore, prebiotic efficiency of parent blueberry polyphenols such as anthocyanins can be maximized through microencapsulation, consequently thwarting degradation from the upper gastrointestinal tract [45]. The health benefits of blueberries are not limited to bioactive polyphenols; a typical serving of blueberries contains large amounts of vitamin C and E, among the major antioxidants in cutaneous systems [46]. Blueberries are also rich in retinol and vitamin A, which have known beneficial effects in the skin [46,47].

## 5. Topical Application

Rigorous research on topical application of natural bioactives suggests their ability to supplement depleted levels of skin micronutrients induced through environmental stressors. Application of compounds containing vitamin E and C successfully replenished the depleted cutaneous antioxidants stores due to O_3_ and UV exposure [49,50,51,52]. The topical application of resveratrol, from the stilbenoid group of polyphenols found in blueberries, can protect against UV- and H_2_O_2_-induced cell apoptosis via SIRT1 activation [53,54]. Prior studies of micronutrient/antioxidant topical supplementation indicate that blueberries are an effective topical defense to common environmental insults as they contain these compounds. Current literature involving direct topical application of blueberry-derived extracts and phytonutrients has demonstrated a reduction in skin inflammation in skin models exposed to O_3_ and UV light [55,56]. Pretreatment with blueberry extract successfully mitigated the deleterious effects on keratinocyte proliferation and migration from O_3_ exposure [55]. Human dermal fibroblasts treated with blueberry anotcynains exhibited reduced NFkB activation and mitigated collagen degradation when exposed to UVB radiation [56]. In 3D cell models such as reconstructed human epidermis (RHE) and ex vivo skin explants, pretreatment with blueberry extract prevented the induction of inflammatory pathways. Blueberry extract was able to reduce the increase in 4-HNE (lipid peroxidation product) and NLRP1, an inflammasome isoform that regulates the activation of caspase-1 which induces an inflammatory response in the epidermis, proven to increase in response to O_3_ exposure [55]. Similar effects were observed in skin models exposed to UV radiation. The pretreatment of blueberry extract on ex vivo skin explants averted UV-induced damage caused through heightened oxidative stress and inflammatory mechanisms [57].

The increase in pro-inflammatory markers 4-HNE and COX-2, regulated through the redox transcription factor nuclear factor kappa-light-chain-enhancer of activated B cells (NF-κB) pathway, was mitigated through pretreatment using blueberry extract on skin exposed to UV light [57,58]. Nuclear factor erythroid 2-related factor 2 (Nrf2), which is another known cellular defense pathway also present in cutaneous tissues, increased in response to UV light, as measured by levels of heme oxygenase-1 (HO-1), an Nrf2-target gene [59]. Skin pretreated with blueberry extract was able to significantly decrease UV-induced HO-1 levels, suggesting the ability to modulate the Nrf2 pathway [57]. Aside from the decrease in inflammatory mechanisms, the topical application of blueberry extract also improves skin barrier function. Exposure to UV light has induced loss of cutaneous structural proteins filaggrin and involucrin. Filaggrin binds keratin fibers to epithelial cells, forming tight bundles which flatten and strengthen cell structure increasing barrier function; involucrin facilitates the differentiation of the keratinocytes in the stratum corneum [60]. The loss of these proteins is directly associated with the development of the chronic skin inflammatory conditions atopic dermatitis and ichthyosis vulgaris [61]. Topical treatment with blueberry extract improves filaggrin and involucrin levels in skin exposed to UV light, which further supports the cutaneous benefits associated with the topical application of blueberries on the skin [57]. In a 12-week clinical study involving the topical application of blueberry extract on hand, arm, and facial skin of type II diabetic females, skin wrinkle formation, smoothness, and moisturization displayed significant improvements [62]. The topical role of particular bioactive compounds, especially polyphenols, present in blueberry extract have yet to be determined, unveiling the need for further studies to elucidate the contributions/physiological mechanisms of these compounds.

## 6. Dietary Supplementation

Dietary supplementation with blueberries is associated with a variety of health-promoting properties and benefits such as increased vascular function, as well as reduced inflammation and oxidative stress. Park et al. demonstrated the ability of blueberry supplementation to decrease the inflammatory markers IL-6 and C-reactive protein (CRP) induced during exercise [63]. IL-6 is a cytokine that contributes to defense through the stimulation of immune reactions [64]. Nieman and colleagues demonstrated a significant post-exercise reduction in plasma levels of arachidonic acid-cytochrome P450-generated oxylipins, a group of metabolites generated through the oxidation of polyunsaturated fatty acids that are involved in inflammation, following a 2-week supplementation of blueberries [65,66]. Systematic reviews provide significant evidence of the ability of blueberries to potentiate a variety of pathways related to vascular function; contrarily, there are conflicting results on the ability of blueberries to reduce inflammation and oxidative stress [65,67,68,69]. In a recent systematic review on oxidative stress, inflammation, and cardio/vascular function modulation by blueberry, Martini et al. cited 45 different acute and chronic human studies, none of which directly measured oxidative and inflammatory markers in the skin or cutaneous properties such as transepidermal water loss (TEWL), skin elasticity, and hydration [67]. This demonstrates the demand for studies involving skin health and blueberry dietary consumption. While the review lacked data directly pertaining to cutaneous effects of blueberry supplementation, it did suggest blueberry supplementation improved overall vascular function [67]. Psoriasis and hidradenitis suppurativa are directly associated with metabolic syndrome and cardiovascular disease, suggesting blueberry supplementation could attenuate these skin conditions [70]. Regarding inflammatory and oxidative markers, the systematic review concluded conflicting results and suggested the need for more studies which are able to provide more evidence in supporting the effect of dietary blueberries intake to counteract oxinflammatory conditions [67]. There are very few in vivo dietary studies with blueberries pertaining *directly* to skin health and the majority utilized mice models; there are even fewer clinical studies on the effect of dietary supplementation on skin health. Park and colleagues demonstrated the ability of fermented black rice and blueberry with *Lactobacillus plantarum* (FBBBR) to increase skin hydration and barrier function after 8 weeks of dietary supplementation in UVB-irradiated hairless mice [71]. In addition, other studies have reported lower skin roughness and increased elasticity in a double-blind 12-week supplementation of Evelle^®^, a skin nutrition supplement with active ingredients of blueberry extract, vitamins C and E, carotenoids, selenium, zinc, amino acids, and glycosaminoglycans [72].

The bioactivity of blueberry polyphenols in physiological cutaneous systems is often questioned due to their purported poor bioavailability directly after ingestion. As aforementioned, dietary polyphenols from blueberries undergo enzymatic modification in the small intestine and are catabolized by the microbiota in the large intestine. After breakdown in the microbiome, a range of phenolic metabolites are absorbed into circulation and are available to human therapeutic targets. Any *individual* polyphenolic metabolite circulates in the blood plasma at nanomolar (nM) concentration; the sum of polyphenol metabolites, depending on level of dietary supplementation, can vary in concentration from 0.1 to 10 micromolar (µM) [73]. Despite their low concentrations, oral supplementation of polyphenolic compounds such as epigallocatechin-3-gallate (EGCG), a stilbenoid derived from in blueberries, has continued to demonstrate cutaneous benefits. Jeon et al. found the regular dietary intake of EGCG in hairless rats strengthened skin tolerance through the attenuation of UV-induced perturbation of the epidermal barrier [74]. 

While the current literature has a plethora of in vitro and animal model studies involving blueberry extracts and individual blueberry-derived compounds, further investigation using clinical trials and physiologically relevant 3D cell models need to be conducted to demonstrate the ability of blueberry consumption to successfully modulate and improve skin functions. The use of physiologically relevant models augments the ability to understand physiological tissue responses when real metabolites concentrations are used, to avoid the overestimation of in vitro and ex vivo results. Therefore, maintaining physiological concentrations in the lab models is imperative for determining the effective mechanisms involved in blueberries’ beneficial effects.

The dietary consumption of blueberries can also modulate the gut microbiome, which plays an important role in a variety of skin disorders (gut–skin axis). An altered or imbalanced skin and gut microbiome have been associated with atopic dermatitis, psoriasis, acne vulgaris, dandruff, and skin cancer [75]. It is known that the increased presence of certain bacteria in the skin microbiome can be distinctly linked to individual skin ailments. For instance, individuals with atopic dermatitis often have higher levels of *Clostridium* and *Faecalibacterium prausnitzii* present on the skin [76,77]. However, this is also true in the gut microbiome. In patients with rosacea, a long-term inflammatory skin condition, *Acidaminococcus* and *Megasphaera* were significantly more abundant in the gut microbiome compared to patients without [78]. Psoriasis patients displayed lower abundance of Bacteroidetes and higher Firmicutes in gut microbiota compared to healthy subjects [79].

It is important to note this is a bidirectional mechanism, as the skin is also able to modify gut microbial composition. In a clinical study, females exposed to UVB light showed decreased serum vitamin D levels, which directly modified gut microbial composition [80]. Blueberry consumption has shown the ability to shape gut microbiota profiles through the increase in *Adlercreutzia equolifaciens* and *Akkermansia muciniphila* in mice [81]. *Akkermansia muciniphila* is a gut microbiota signature in psoriasis, as recent studies demonstrate the abundance of this bacterium is significantly *reduced* in afflicted patients [82]. The successful increase in *Akkermansia muciniphila* through blueberry consumption suggests a possible intervention in psoriasis patients. In addition, wild blueberry extract-fed mice also demonstrated an increase in the bacterial family *Verrucomicrobiaceae*, known to be decreased in the gut microbiota of psoriasis patients [81,83]. This is another example of how blueberry consumption modifies microbiota profiles whose alteration may be able to improve symptoms of skin diseases. Furthermore, Vendrame et al. reported an increase in bifidobacteria in the human gut following six-week consumption of a blueberry drink [84]. Bifidobacteria are commonly used in probiotics; a recent study reveals numerous benefits of probiotics containing bifidobacteria in plaque psoriasis patients such as decreased inflammatory markers CRP and IL-6 [85]. Following the daily 6-week consumption of freeze-dried blueberries, *Faecalibacterium prausnitzii* (FP), *Barnesiella intestinihominis*, *Eubacterium halii* (EH), *Anaerostipes hadrus*, and *Ruminococcus bromii* (RB) levels were abundant compared to the baseline measurements [86]. FP, EH, and RB are dominant intestinal bacterial species associated with numerous benefits through their production of the short-chain fatty acid butyrate, which functions to decrease oxidative stress and provide anti-inflammatory effects in the gut [83]. Butyrate is also a direct source of energy for gut epithelium which further provides benefits. Low levels of FP have been identified in the skin diseases psoriasis, atopic dermatitis, and scleroderma; additionally, *Ruminococcus* genus dysbiosis has been demonstrated in psoriasis and psoriatic arthritis patients [76,83,87,88,89]. The ability of blueberry supplementation to increase these bacteria associated with a variety of skin ailments effectively highlights their possible usage as an intervention or prevention strategy. Further elucidation of gut profiles associated with skin disorders and blueberry microbiome modification are needed to confirm this symbiotic mechanism; however, current literature suggests blueberry consumption can effectively modify human gut microbiota profiles, which can likely provide benefits in cutaneous systems.

## 7. Concluding Remarks

The present review summarizes the potential health benefits of blueberries and how they can be recruited topically and dietarily to improve skin functions and prevent/protect from environmental damage. Skin health depends on both extrinsic agents and intrinsic factors. The intrinsic aging of the skin is a natural consequence of physiological changes over time due to genetic predisposition, whereas extrinsic aging is a consequence of exposure to environmental factors and lifestyle. Skin aging can be modulated by both dietary intervention and topical application. Indeed, GI tract and skin have different ways to absorb nutrients, leading to the activation of cellular defensive systems by different mechanisms. Dietary intervention alone is not enough to prevent/treat skin conditions due to skin anatomy, as the epidermis does not receive blood supply, and, therefore, there is no direct delivery of nutrients to the epidermis. Utilizing both topical applications and dietary intervention is needed for optimal nutrient delivery to the skin. We believe that utilizing this two-pronged approach to optimize antioxidant protection will decrease environmentally induced oxinflammation and the development/exacerbation of premature aging.

Nonetheless, it is necessary to elucidate the mechanisms involved in the beneficial causes of blueberries on cutaneous systems. The roles of many blueberry-derived metabolites present in the bloodstream are still unknown. More research needs to examine the role of the blueberry-derived metabolites found in blood plasma and their ability to modulate cutaneous mechanisms (enzymatic functions, antioxidant defense, inflammasome activation). Research on the amount of time these metabolites are present systematically will help to clarify how long benefits to cutaneous systems can be expected. See Table 2.

## Figures and Tables

**Table 1:** Adapted from Rodrigues-Mateos et al. List of top 5 blueberry metabolites upregulated through ingestion of a blueberry beverage. Values are maximum nanomolar concentration of metabolite obtained in blood plasma [48].

Metabolite	Concentration in Plasma (nM)
**Hippuric acid**	5456 ± 1274
**α-Hydroxyhippuric acid**	4322 ± 1607
**Benzoic acid**	385 ± 16
**Syringic acid**	241 ± 201
**3-and 4-Hydroxyphenylacetic acid**	241 ± 50 327 ± 22
**Hydroxycinnamic acids (Ferulic, Dihydroferulic, Isoferulic)**	194 ± 2 256 ± 52 217 ± 44

**Table 2:** Current literature articles involving blueberries and cutaneous health with a summary of findings.

Authors	Model	Findings
**Roy, S. et al., 2002 [56]**	In vitro	Blueberry extract-treated human HaCaT keratinocytes displayed significant reductions in hydrogen peroxide and TNF⍺-induced vascular endothelial growth factor (VEGF) expression.
**Wang, H. et al., 2019 [90]**	In vitro	On human HaCaT keratinocytes and human foreskin fibroblast cells exposed to UV-C radiation, blueberry extract exhibited protective effects: decrease DNA fragmentation, inhibited MMP-1 and other inflammatory factors expression.
**Hong, S. et al., 2021 [91]**	In vitro/In vivo	Fermented blueberry and black rice (FBBBR) extract mitigated particulate matter-induced inflammation in HaCaT cells through proinflammatory cytokine reduction (IL-1β, IL-6, IL-8). FBBBR improved transepidermal water loss, decreased skin erythema, AD-like symptoms, cytokines (IFN-γ, IL-2, IL-4, and IL-10), and scratching tendency in mice exposed to particulate matter.
**Grether-beck, S, et al., 2017 [92]**	In vitro	Blueberry-derived antioxidant matrix (Berrimatrix™) successfully prevented infrared A-induced MMP-1 expression in primary human skin fibroblasts.
**Bae, J. et al., 2009 [93]**	In vitro	Bog blueberry extract attenuated UV-B triggered collagen damage and release of IL-6 and IL-8 in human dermal fibroblasts.
**Yamasaki, S. et al., 2018 [94]**	In vitro	Normal human epidermal keratinocytes pretreated with lowbush blueberry extract exhibited significant attenuation in UV-B-induced damage.
**Park, S. et al., 2021 [71]**	In vivo	In UVB-irradiated hairless mice, FBBBR oral supplementation improved stratum corneum hydration, epidermal thickness, and increased filaggrin, involucrin, TGM-1, and COL1A1 mRNA expression in skin.
**Pambianchi, E. et al., 2020 [55]**	In vitro/Ex vivo	Pretreatment with blueberry extract displayed protective effects in HaCaT, reconstructed human epidermis, and human skin explants exposed to ozone. Blueberry extract enhanced keratinocyte wound closure and prevented O_3_-induced ROS production and inflammasome activation (reduction of CASP1, IL-18). Blueberry extract also prevented O_3_-induced increase in 4-HNE, ASC, and NLRP1 in human skin explants.
**Pambianchi, E. et al., 2021 [57]**	Ex vivo	Topical application of Alaskan bog blueberry extract prevented UVA/UVB-induced 4-HNE, HO-1, COX2 expression and decreased the loss of cutaneous structural proteins (filaggrin and involucrin) in human skin explants.
**Draelos, Z.D. et al., 2009 [62]**	Clinical	/Topical application of blueberry extract-containing product for 12 weeks increased cutaneous moisturization, smoothness, radiance, and firmness in type II diabetic skin.
**Segger, D. et al., 2004 [72]**	Clinical	Dietary supplementation of Evelle (a blueberry extract-containing product) for 12 weeks successfully improved skin elasticity and reduced skin roughness.
**Schiavon, D. et al., 2019 [95]**	Clinical	Blueberry extract- and microparticles-formulated sunscreen exhibited increased photoprotective capacity in the stratum corneum of humans exposed to UV light.

## Data Availability

Data is contained within the article or supplementary material. The data presented in this study are available in [48].
